# Hémangiopéricytome de l'angle ponto-cérébelleux: cas clinique et revue de la littérature

**DOI:** 10.11604/pamj.2015.20.61.4198

**Published:** 2015-01-22

**Authors:** Jaafar Najib, khalid Aniba, Mehdi Laghmari, Mohammed Lmejjati, Houssine Ghannane, Said Ait Benali, Hind Ennadam, Hind Jalal, Cherif idrissi

**Affiliations:** 1 Service de Neurochirurgie, Hôpital Ibn-Tofail, CHU Mohammed VI, Marrakech 40000, Maroc; 2 Service de Radiologie, Hôpital Ibn-Tofail, CHU Mohammed VI, Marrakech, Maroc

**Keywords:** Angle ponto-cérébelleux, hémangiopéricytome, IRM, chirurgie, cerebellopontine angle, Hemangiopericytoma, MRI, surgery

## Abstract

Les hémangiopéricytomes primitifs du système nerveux central sont rares et représentent moins de 1% des tumeurs intracraniennes. La localisation au niveau de l'angle ponto-cerebelleux est très rare, pouvant simuler un neurinome de l'acoustique ou un méningiome. Le diagnostic de certitude est basé sur l’étude histologique et immunohistochimique. Notre but est d'illustrer avec une revue de la littérature les aspects clinico-radiologiques, anatomopathologiques et la prise en charge thérapeutique de ce type de lésion.

## Introduction

L'hémangiopericytome est une tumeur mésenchymateuse rare, développée aux dépens des pericytes de Zimmerman: cellules contactiles entourant les capillaires. Elle a été décrite pour la première fois en 1942 par A.P. Stout et M. Murray [1]. Elle représente moins de 1% des tumeurs intracrâniennes. Son siège au niveau de l′angle ponto-cérébelleux ainsi que son aspect en tomodensitométrie ou en imagerie par résonance magnétique peut être trompeur et faire porter à tort le diagnostic de neurinome de l′acoustique ou de méningiome. L'hémangiopericytome se caractérise par son potentiel malin, son taux élevé de récidive et de métastases à distance, justifiant une exérèse chirurgicale large et une radiothérapie complémentaire. Nous rapportons un cas d'hémangiopéricytome méningé de l′angle ponto-cérébelleux, très rare qui constitue un piège diagnostic vu la fréquence du neurinome de l′acoustique et du méningiome au niveau de l′angle ponto cérébelleux.

## Patient et observation

**Figure 1:**
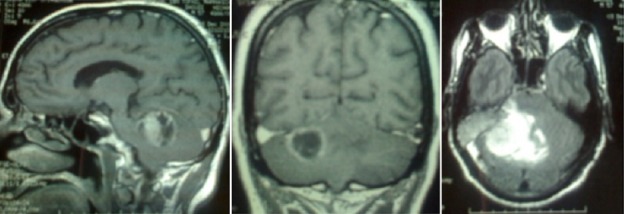
IRM encéphalique objectivant la présence processus expansif de l'angle ponto-cerebelleux étendu au rocher mesurant 20 mm×30 mm, hyperintense en T1 et T2, se rehaussant de façon hétérogène après l'injection du produit de contraste avec effet de masse sur le V4

**Figure 2:**
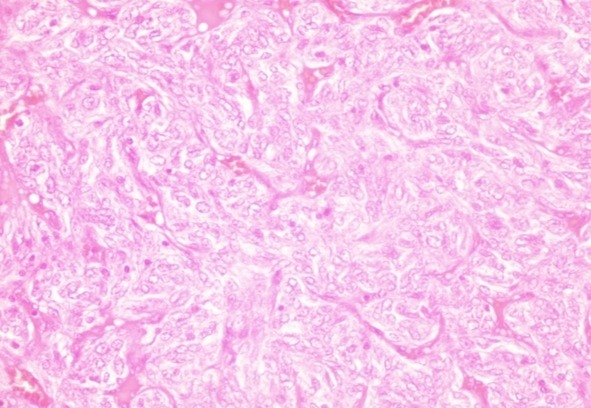
L'aspect anatomopathologique de la tumeur après coloration standard, montre un processus tumoral mal limité, d'aspect multinodulaire qui se caractérise par une densité cellulaire élevée, faite de nappes cellulaires peu cohésives. La vascularisation est riche, réalisant un aspect angiomateux. Cette vascularisation est spéciale, caractérisée par un aspect ramifié, souvent en « bois de cerf ». La lumière vasculaire n'est pas toujours visible. L'anisocaryose est modérée. L'activité mitotique est élevée atteignant par places 4 mitoses / champ au fort grandissement

Monsieur H.D., âgé de 58 ans, sans antécédents pathologiques particuliers, est hospitalisé pour un syndrome d'hypertension intracranienne fait de vomissements et céphalées, rebelles au traitement antalgique, associées à des vertiges, bourdonnement d'oreille, troubles de l’équilibre et une diplopie. L'examen clinique trouve un patient conscient, sans déficit neurologique apparent avec des réflexes ostéotendineux conservés. L'IRM encéphalique objective la présence d'un processus expansif de l'angle ponto-cerebelleux étendu au rocher mesurant 20 mm×30 mm, hyperintense en T1 et T2, se rehaussant de façon hétérogène après l'injection du produit de contraste avec effet de masse sur le V4 (Figure 1). L'image radiologique a fait discuter comme diagnostic, un neurinome ou un méningiome de l'angle ponto-cérébelleux. Le patient est opéré, le geste opératoire a consisté en une résection large. L’étude anatomo-pathologique (Figure 2) après coloration standard, montre un processus tumoral mal limité, d'aspect multinodulaire qui se caractérise par une densité cellulaire élevée, faite de nappes cellulaires peu cohésives. La vascularisation est riche, réalisant un aspect angiomateux. Cette vascularisation est spéciale, caractérisée par un aspect ramifié, souvent en « bois de cerf ». La lumière vasculaire n'est pas toujours visible. L'anisocaryose est modérée. L'activité mitotique est élevée atteignant par places 4 mitoses/champ au fort grandissement. L’étude immunohistochimique a trouvée un marquage intense avec le CD31 et le CD34 qui réhausse le réseau vasculaire alors que le marquage par l'EMA a été négatif. Elle a conclue au diagnostic d'hémangiopéricytome méningé.

## Discussion

Les hémangiopéricytomes du système nerveux central sont rares: ils représentent moins de 1% des tumeurs intracraniennes et 2 à 2,4% des tumeurs méningées [2, 3]. La localisation au niveau de l′angle ponto-cerebelleux est très rarement rapportée dans la littérature [4, 5]. A la différence des méningiomes, les hémangiopéricytomes ont une prédilection pour le sexe masculin [6, 7]. L’âge moyen de début variant de 38 à 42 ans [8, 9]. Leur distribution est similaire à celle des méningiomes [10, 11]. Ils siègent essentiellement à l′étage sus-tentoriel, volontiers parasagittaux au contact des sinus veineux [5], 77% dans la série rapportée par Jeong-Hoon et al., 2003 et 66% dans celle de Dufour et al., 2001. Il est fréquent que l′imagerie (TDM, IRM) ne permet pas de porter le diagnostic d′hémangiopéricytome et de le différencier d′un méningiome [12]. Cependant, certains éléments orientent vers le diagnostic d′hémangiopéricytome: les calcifications sont exceptionnelles. Il est typiquement hétérogène avant et après contraste [12, 13]. Le rehaussement est souvent intense après injection de PDC. La base d′implantation est souvent large, parfois pédiculée ce qui est inhabituel pour un simple méningiome. Une érosion ou une lyse osseuse sont classiques mais aspécifiques. Il n′existe jamais d′hyperostose pour les hémangiopéricytomes. Contrairement aux méningiomes, l′hémangiopéricytome s′accompagne d′une infiltration locorégionale importante [4, 12]. Parmis ces critères, l′imagerie de notre patient a montrée un réhaussement important après injection du PDC, une base d′implantation pédiculée avec une atteinte osseuse. A l′angiographie, les hémagiopéricytomes se caractérisent par: un apport artériel souvent mixte, par des branches à destinée parenchymateuse (carotide interne surtout et artère vertebrale) et par des vaisseaux d′origine méningée. La vascularisation est anarchique (aspect en tire bouchon des vaisseaux tumoraux). Le blush tumoral est d′abord intense et homogène et persiste de façon hétérogène, témoignant d′un ralentissement circulatoire intratumoral [12]. Cependant le diagnostique de certitude est basée sur l′étude anatomopathologique et immunohistochimique; les cellules tumorales sont marquées par les anticorps dirigés contre le CD 34 mais négative pour les anticorps anti-facteur VIII et la proteine S-100 [5, 12]. Le traitement repose sur l′exérèse complète de la tumeur, suivie d′une radiothérapie du fait du potentiel de récidive et de métastase à distance très élevé [8, 10]. Toutefois, la nature très hémorragique de la tumeur ainsi que son siège peuvent poser des problèmes majeurs d′émostase et rendre l′exérèse délicate ce qui a été le cas de notre patient. La mortalité opératoire varie de 0 à 27% et certains décès sont secondaires au saignement, raison pour laquelle certains auteurs recommandent l′embolisation prè-opératoire lorsque le diagnostic d′hémagiopéricytome est suspecté [8]. Les hémangiopéricytomes sont plus radiosensibles que les méningiomes. Initialement réservée aux tumeurs non résécables ou métastatiques, la radiothérapie est désormais réalisée quasi systématiquement en complément de la chirurgie [4]. De nombreuses études ont montré que la radiothérapie post-opératoire diminue significativement le pourcentage de récidives et améliore le pronostic des patients. Dufour et al [8] rapportent des taux de récidive de 12% dans le groupe de patients irradiés versus 88% dans le groupe de patients non irradiés. Guthrie et al [14] rapportent des taux de récidive de 52% chez les patients irradiés versus 86% chez les patients non irradiés. Le taux de récidive locale varie de 26% à 80% [8, 9], il dépend de la qualité de l′exérèse, de la durée de suivi et de la pratique d′une radiothérapie postopératoire. Dufour H et al rapportent un taux moyen de récidive de 45%. Il est de 38,7% pour Jeong HoonK et al. La majorité de ces récidives surviennent localement, au niveau du site tumoral initial et plus rarement à distance [9]. Les localisations métastatiques les plus fréquentes sont l′os, le poumon et le foie [14]. La probabilité de survenue de métastases augmente avec le temps: Guthrie et al rapportent des taux à 5,10 et 15 ans respectivement de 13%, 33% et 64%. Jeong-Hoon et al rapportent des taux à 5 et 10 ans respectivement de 4,4% et 24,9%. Ceci impose une surveillance stricte à long terme basée sur un examen clinique complet, une radiographie du thorax, une échographie abdominale ou un pet scan lorsqu′il est indiqué [8, 9].

## Conclusion

L'hémangiopéricytome de l'angle ponto-cérébelleux est très rare. Le traitement repose sur la chirurgie et la radiothérapie. Ce sont des tumeurs à haut potentiel de récidive et de métastase, imposant un suivi prolongé.
